# Clay-Facilitated Aqueous Dispersion of Graphite and Poly(vinyl alcohol) Aerogels Filled with Binary Nanofillers

**DOI:** 10.3390/gels4010008

**Published:** 2018-01-12

**Authors:** Lei Liu, Yuxin Wang, Saeed M. Alhassan, Hua Sun, Kyungwho Choi, Choongho Yu, David A. Schiraldi

**Affiliations:** 1 Department of Macromolecular Science and Engineering, Case Western Reserve University, Cleveland, OH 44106-7202, USA; yuxin0108@hotmail.com (Y.W.); hxs344@case.edu (H.S.); 2 Department of Chemical Engineering, The Petroleum Institute, P.O. Box 2533, Abu Dhabi, United Arab Emirates (UAE); salhassan@pi.ac.ae; 3 Department of Mechanical Engineering, Texas A&M University, College Station, TX 77843-3123, USA; kwchoi80@gmail.com (K.C.); chyu@tamu.edu (C.Y.)

**Keywords:** polymer aerogels, clay, aqueous dispersion, mechanical properties, electrical properties

## Abstract

Dispersion of graphite in water was achieved using clay as dispersing aid. In the absence of polymer, the clay/graphite suspensions were sufficiently stable to produce aerogels composed of very thin layers of uniformly dispersed nanoparticles. Poly(vinyl alcohol) (PVOH) aerogels containing binary nanofillers (clay plus graphite) were then fabricated and tested. These composites were found to maintain low thermal and electrical conductivities even with high loading of graphite. A unique compressive stress-strain behavior was observed for the aerogel, exhibiting a plateau in the densification region, likely due to sliding between clay and graphite layers within the PVOH matrix. The aerogels containing only graphite exhibited higher compressive modulus, yield stress and toughness values than the samples filled with binary nanofillers. X-ray diffraction (XRD) spectra for the same composite aerogel before and after compression testing illustrated the compression-induced dispersion changes of nanofillers. Composites containing 50 wt % graphite demonstrated a downshift of its 2D Raman peak implying graphite exfoliation to graphene with less than 5 layers.

## 1. Introduction

Aerogels are known for their ultralow densities and exceptionally low thermal conductivities, which arise from inclusion of high volume fractions of air in their structures. These cellular structures, together with large surface areas, lead to applications such as thermal or acoustic insulation, packaging, catalyst support and structural materials. Since the first report in 1931 by Kistler [1], silica aerogels have been extensively studied and formation of these materials typically involves sol-gel processes with solvent removal by supercritical drying [2,3,4]. Clay aerogels, however, can be more conveniently fabricated through gelation when clay concentrations in aqueous suspension exceed their critical gelling concentration, with subsequent removal of water by freeze drying [5]. House-of-cards structures with bulk densities ranging from 0.05 to 0.15 g/cm^3^ are typically found for such aerogels, which are very fragile due to the weak interactions between clay clusters within layered structures, preventing their use in practical applications. Water-soluble or water-dispersible polymers are therefore introduced to the clay gels to improve mechanical rigidity and integrity through the widely accepted improvement of load transfer characteristics for clay-filled polymer composites, which maintain good processibility simultaneously [6,7,8].

Graphite is composed of graphene sheets stacked on top of each other with an interlayer distance of 3.37 Å. Single layered graphene is found to be among the strongest materials ever measured, with a Young’s modulus and ultimate tensile strength of 1 TPa and 130 GPa, respectively. The combination of robust mechanical properties and superb thermal and electrical conductivities makes graphene an ideal performance-enhancing additive for polymers. Similar to carbon nanotubes, which are difficult to exfoliate due to strong van der Waals attractions [9], various techniques have been developed to produce high quality graphene sheets from graphite. Graphene oxide (GO) is one of the promising methods, wherein graphite is exposed to strong oxidants under acid conditions to create epoxide and hydroxyl functional groups on graphene layers [10]. However, it is electrically insulating, thermally unstable and found to contain large percentage of oxygen [11]. Other approaches that produce graphene from graphite include micromechanical cleavage [12], sonication in organic solvents [13], and electrochemical exfoliation [14], whereas little attention has been paid to directly disperse graphite into multi-layer graphene under the help of inorganic surfactants in water [15].

In the current study, clay was used as surfactant to stabilize graphite in water. The mixture was stable at ambient conditions for weeks, and clay/graphite aerogels and their poly(vinyl alcohol) (PVOH) composite aerogels were subsequently fabricated. This method provides a simple yet effective way to disperse graphite into multi-layer graphene and incorporate into aqueous suspension and gel, which leaves the intrinsic properties of graphite intact. PVOH composites containing clay and graphene were reported to exhibit improved mechanical properties due to synergistic reinforcement using in situ reduced GO [16]. However, the current approach aims to adopt a different processing technique (freeze-drying) to make graphene-filled aerogels without the introduction of GO, oxidative reagents and high temperature. The introduction of binary fillers offers enhanced dispersibility and larger selection of materials for multifunctional composites with potential applications such as batteries and supercapacitors.

## 2. Results and Discussion

**Figure 1:**
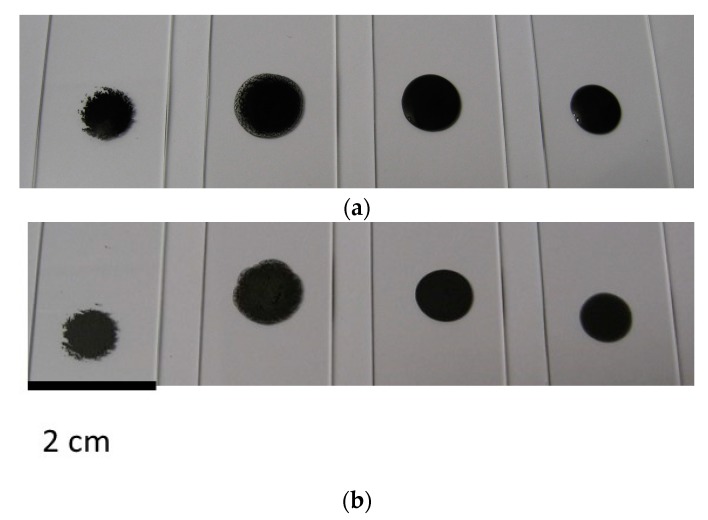
Digital images for liquid drops of clay/graphite aqueous suspensions on glass slides before (**a**); and after drying (**b**), for clay/graphite ratios of 0, 1:4, 1:1, 4:1 (from left to right).

Without any polymer addition, aqueous suspensions containing various ratios of clay to graphite were prepared and the total solid concentrations were kept at 5 wt %. Digital images for representative liquid drops right after preparation are shown in Figure 1a. When the ratio of clay to graphite is 0 (only 5 wt % graphite in water without any clay), the liquid remained transparent and highly aggregated graphite clusters were observed in the suspension, as expected given the hydrophobic characteristics of pristine graphite. Improved graphite dispersion was observed after the ratio was increased to 1:4, as evidenced by reduced cluster size and grayish color of the liquid. A 1:1 ratio of clay to graphite resulted in a homogeneous black suspension with noticeably increased viscosity. When the ratio reached 4:1, a highly viscous liquid was obtained with the appearance of a stable dispersion of graphite. Precipitation was observed with the 0 and 1:4 ratio suspensions, while the liquid retained its dark color even after a month for the sample with a ratio of 1:4. The suspension with a ratio of 4:1 exhibited faster gelation, relative to the sample with a ratio of 1:1, presumably due to higher clay loading for the former. Upon drying (Figure 1b), the suspensions containing lower clay to graphite ratios (0 and 1:4) left large graphite particles on glass slides, whereas uniform black coating was obtained for samples with higher ratios, indicating improved graphite dispersion could be maintained during drying.

**Figure 2:**
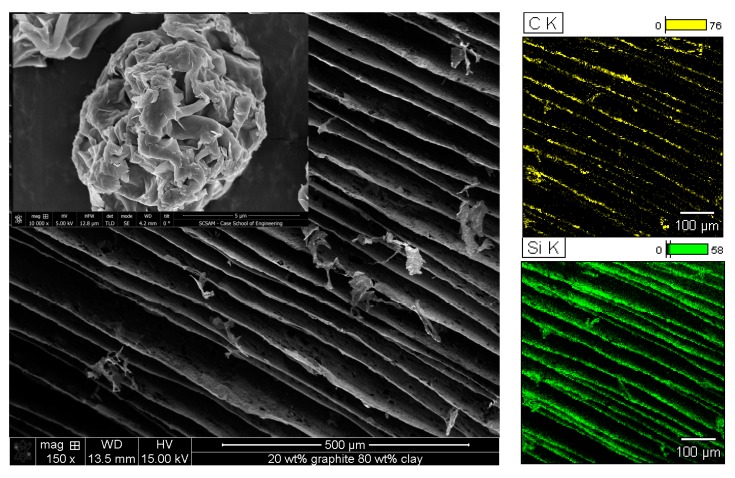
SEM image and elemental mapping analysis for an aerogel containing 20 wt % graphite and 80 wt % clay; the SEM image for pristine clay particles is inserted.

The aqueous suspensions of clay and graphite were then freeze dried to form clay/graphite aerogels. The samples produced with clay to graphite ratio of 1:4 resulted in powders rather than aerogels after drying due to their limited clay content. Aerogel samples were successfully fabricated from suspensions with higher clay to graphite ratios. Figure 2 shows the SEM image and elemental mapping for carbon and silicon for the aerogel produced from suspension with clay to graphite ratio of 4:1, which contains 20 wt % graphite and 80 wt % clay. A layered structure with individual layer thickness and interlayer spacing of approximately 1 µm and 80 µm, respectively, was formed due to the crystalline ice formation during freezing and subsequent sublimation. Elemental mapping of carbon and silicon confirms the uniform distribution of graphite and clay in the lamellar structure.

**Figure 3:**
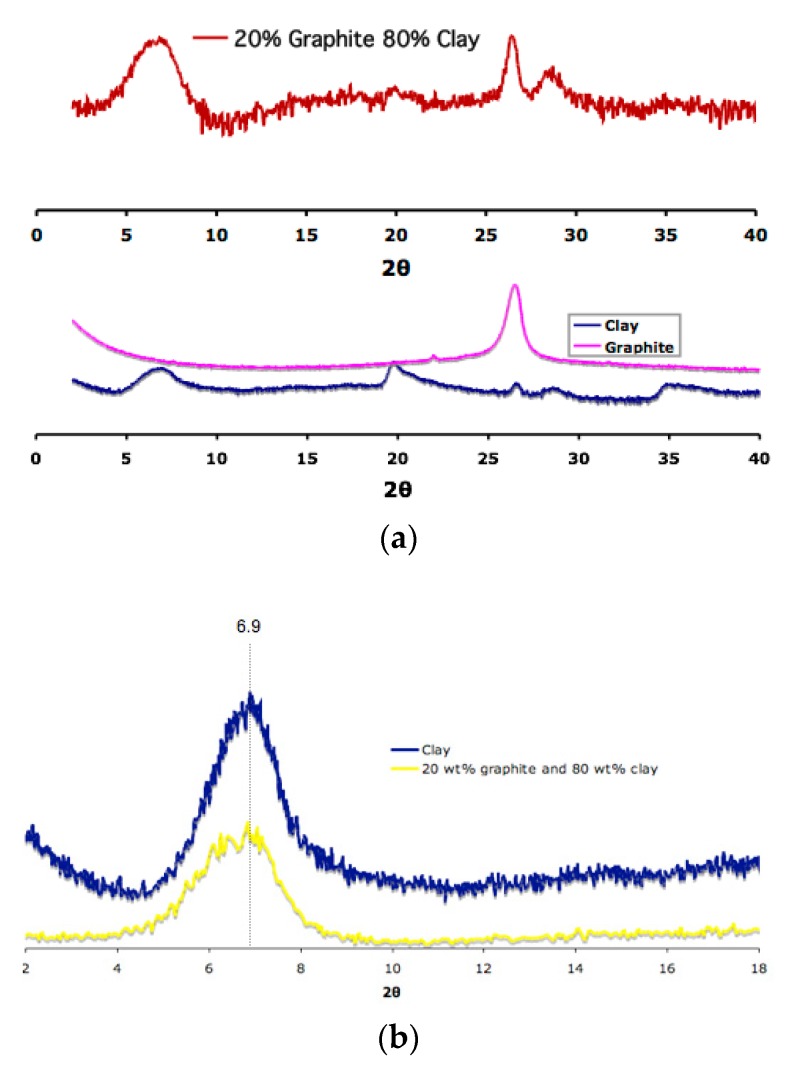
X-ray diffraction (XRD) patterns of pristine clay, graphite, and the aerogel containing 20 wt % graphite and 80 wt % clay (**a**); and clay XRD peaks for pristine clay and the aerogel containing 20 wt % graphite and 80 wt % clay (**b**).

The degree of exfoliation for clay and graphite was determined by X-ray diffraction (XRD), as shown in Figure 3. A diffraction peak at 2θ ≈ 6.9° that corresponds to the (001) plane with spacing of 1.28 nm is characteristic for pristine clay powder. Graphite exhibits 2θ ≈ 26.3° for (002) plane, showing an interlayer distance of 3.38 Å. The aerogel produced from untreated clay and graphite was found to contain both of these peaks, which suggests the presence of stacks of the two nanofillers (Figure 3a). However, it is clear that some of the clay was intercalated (Figure 3b) as evidenced by new peaks at 2θ lower than 6.9° *as previously reported by Morgan and Gilman* [17]. This indicates that by just using graphite as a dispersing aid, clay can be intercalated in a polymer-free process. It should be noted that although conclusive results for graphite exfoliation could potentially be obtained using XRD, this method is only sensitive to strong reflections. For example, the reflective peak for the (002) plane of graphite was found to be still present even when very small amount (0.1%) of graphite was present in the highly oxidized sample of GO, which otherwise would not show the peak [18].

The introduction of graphite into aerogels took place when successful gelation of the two nanofillers in water was achieved. Suspensions with clay to graphite ratio of 4:1 and 1:1 led to aerogels with 20 and 50 wt % of graphite loading, respectively, while maintaining large volume fraction of air. The ease of water processing and possible inclusion of large quantity of graphite may benefit various applications. Poor mechanical properties of these aerogels, however, represent a major hurdle for their direct utilization. Producing polymer composites with clay/graphite mixtures may offer a solution to this problem given that both of these two materials are well known fillers in polymer composites. Clay is mechanically strong and known to show good load transfer characteristics in polymer composites [19]. Addition of clay may benefit other properties such as flame retardation and gas barrier [20,21]. The use of clay as a surfactant to facilitate carbon nanotube dispersion in epoxy has been reported, which exhibited reduced percolation threshold while improving the electrical conductivity and storage modulus, simultaneously [22]. So the combination of both clay and graphite into polymer composites could have great potential to introduce the advantages of two nanofillers into one system.

**Figure 4:**
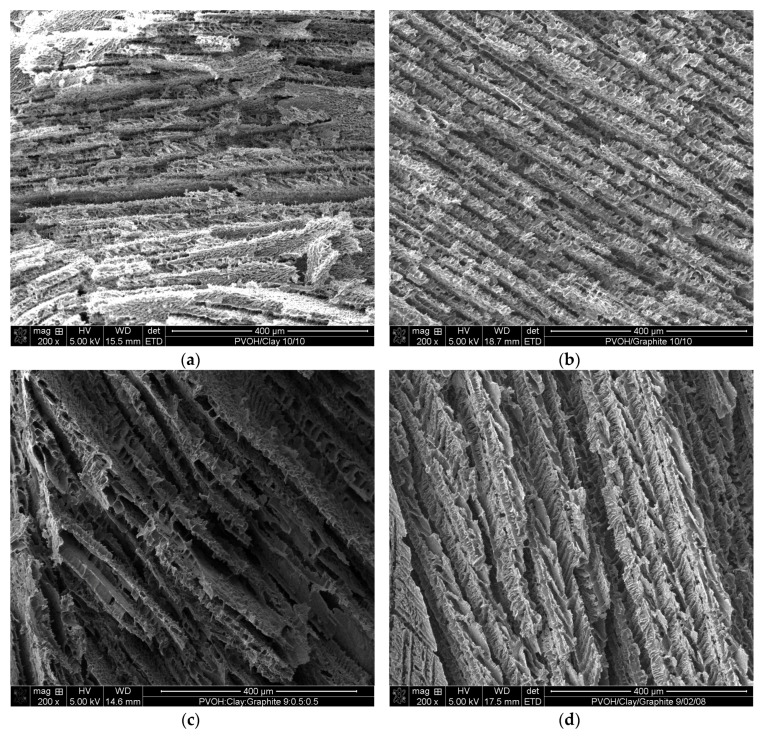

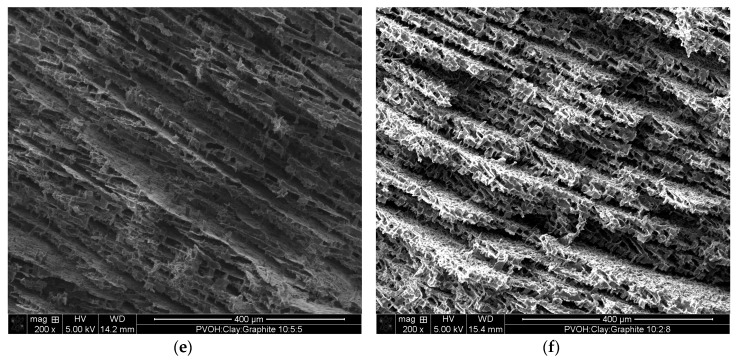
SEM images for PVOH aerogels containing: 50 wt % clay (**a**); 50 wt % graphite (**b**); 5 wt % clay and 5 wt % graphite (**c**); 2 wt % clay and 8 wt % graphite (**d**); 25 wt % clay and 25 wt % graphite (**e**); and 10 wt % clay and 40 wt % graphite (**f**).

Poly(vinyl alcohol) (PVOH) was chosen for this study due to the previous success of making polymer aerogels and good interactions with clay and graphite. Figure 4 shows the SEM images of PVOH aerogels containing different concentrations of clay and graphite. Uniformly layered structures were formed for all of the samples after freeze drying and the resultant lamellar foam-like structures with 50 wt % clay addition (Figure 4a) exhibited similar morphology as reported in literature [8]. Samples containing 50 wt % graphite (Figure 4b) exhibited similar layer thicknesses (about 22 µm) and stacking, compared to the 50 wt % clay samples. When a 10 wt % mixture of clay and graphite was introduced into PVOH aerogels (Figure 4c,d), two different layer thicknesses were observed at 10 and 35 µm for clay/graphite ratio of 1:1 and 1:4, respectively. Layers in some cases appear to be bridged by PVOH to form larger layers. The samples loaded with 50 wt % binary fillers exhibited fairly consistent 35 µm layer thicknesses, as shown in Figure 4e,f, indicating that the layer thickness would be smaller when loaded with single nanofiller (either clay or graphite), compared with binary nanofiller at the same total loading.

**Figure 5:**
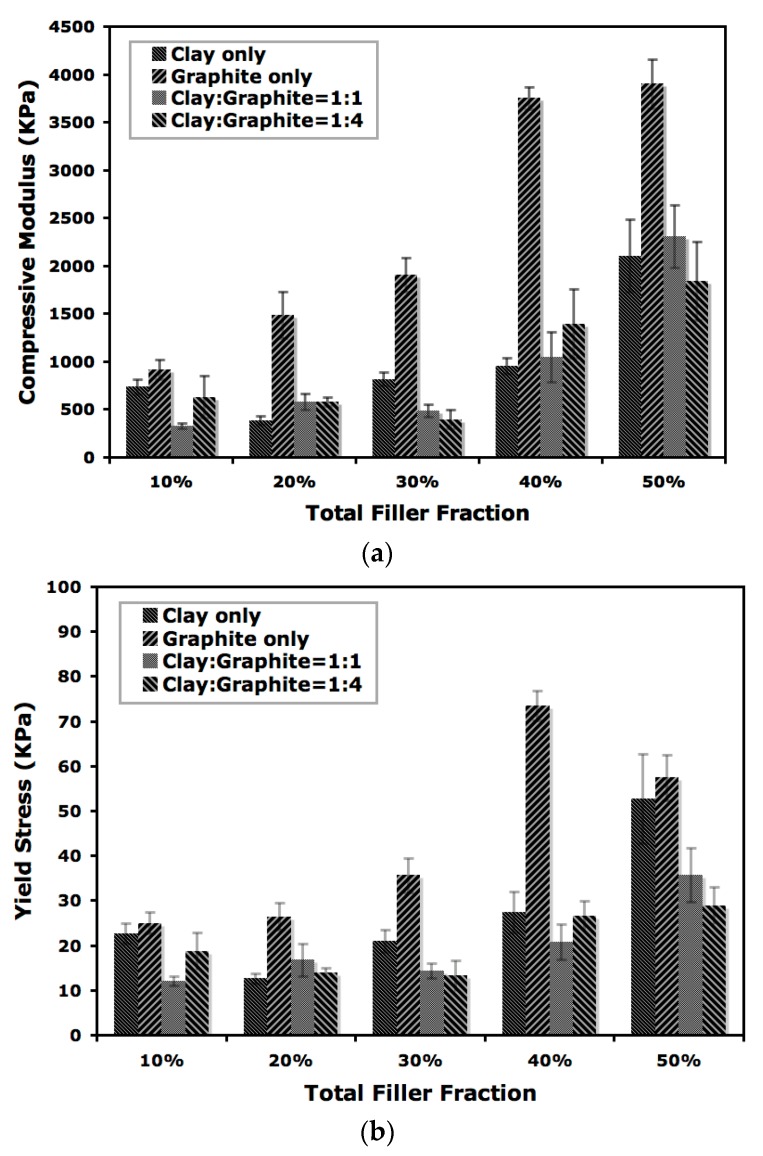
Compressive modulus (**a**) and yield stress (**b**) as a function of total nanofiller loading (in wt %) for PVOH aerogels, mean and standard deviations (*n* = 4) given.

Mechanical performance of the aerogels was evaluated by compression testing and the impact on compressive modulus by total nanofiller loading is shown in Figure 5. Many factors are known to affect the mechanical behavior of polymer aerogels, such as molecular weight, freezing conditions, filler loading and polymer-filler interactions. For the current study when binary nanofillers are introduced into polymer aerogels, the interaction between polymer and both of the two nanofillers in the composites would need to be considered. Moreover, the interaction between the nanofillers will also play an important role in determining the performance of aerogels [15]. A nearly linear increase of compressive modulus with increasing filler loading above 20 wt % was observed for the aerogels produced in this study. Variations in modulus were noticed with smaller loadings of nanofillers, potentially attributable to insufficient formation of a reinforcement network and coexistence of variable layer thickness in the composites. The aerogels reinforced with graphite alone showed the highest (2- to 4-fold) modulus enhancement relative to the composites containing a mixture of clay and graphite or clay only. It is worth noting that the composite containing 40 wt % of graphite showed a compressive modulus of 3.9 GPa, while the addition of extra 10 wt % of clay (total nanofiller loading of 50 wt % with a clay to graphite ratio of 1:4) lowered the modulus to 1.8 GPa, which is close to the modulus for the aerogel containing 30 wt % graphite (1.9 GPa). The coexistence of clay and graphite introduced new interface between nanofillers and while successful load transfer from graphite to aerogel composites can occur, the presence of clay could also prevent contact between PVOH and graphite due to the van der Waals interaction among the two nanofillers, leading to a decrease in load bearing for aerogels loaded with binary nanofillers. Another possible reason for reduced reinforcement when clay and graphite are combined is inhibited formation of initial ice crystals on mold surfaces, which are known to determine the dimension and geometry of lamellar structure for aerogels [23]. The influence on yield stress for composites with different filler concentrations was shown in Figure 5b. A similar trend was observed for the yield stress, although a noticeable decrease was found when graphite loading reached 50 wt %. The reported synergistic effects were not found for the current study [16], possibly due to different nanofiller loading and sample preparation techniques.

**Figure 6:**
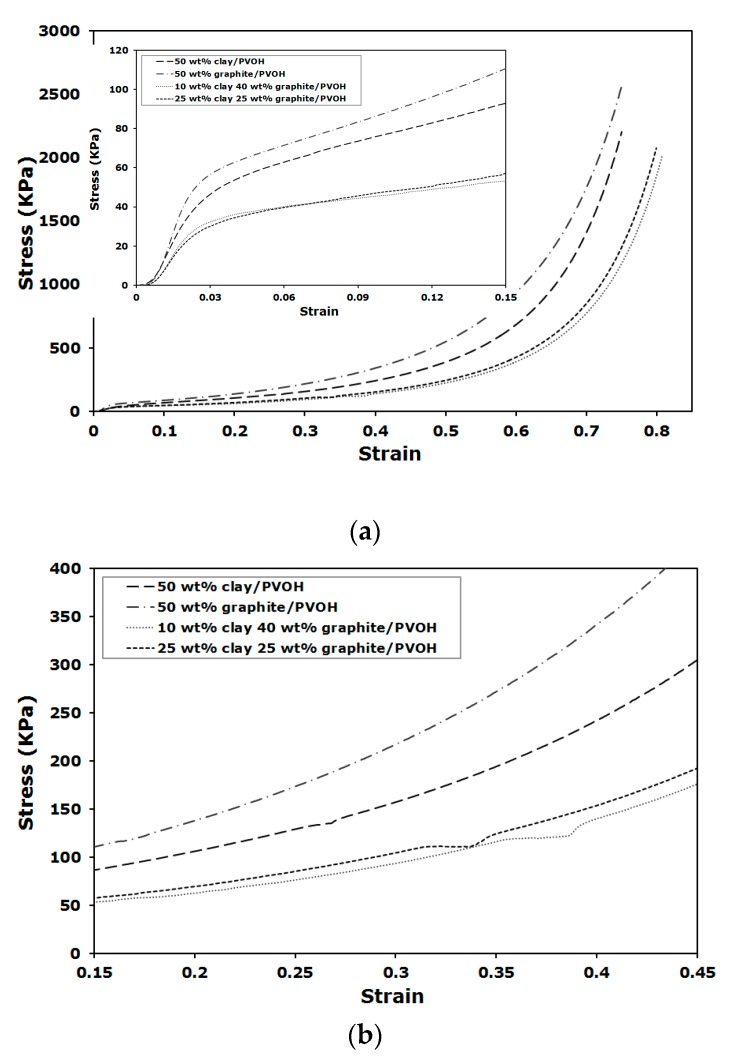
Compressive stress-strain curves for the aerogels loaded with 50 wt % of total nanofiller (**a**) and in the strain range between 15% and 45% (**b**).

Stress-strain curves for the compression tests are shown in Figure 6. The curves show typical compression behavior for rigid porous foam, which starts with a linear elastic deformation at low strain and followed by densification region after yielding when the structures collapse (Figure 6a). Besides the variation in compressive modulus and yield stress, as summarized in Figure 5, the samples loaded with a total amount of 50 wt % nanofiller showed different toughness behavior. The aerogels loaded with 1:1 and 1:4 clay to graphite ratios showed similar toughness, which is defined as the area under the stress-strain curve. Toughness was nearly doubled for the sample containing 50 wt % of graphite, relative to the samples with binary nanofillers, indicating little impact on energy adsorption per unit volume using binary nanofillers at different ratios. The presence of a plateau in the densification region, shown as a discontinuity in the normally monotonically-increasing curve between approximately 30–40% strain, is shown in Figure 6b. This unique behavior is not observed for the several hundred different polymer/clay aerogels we have tested before and during the present study and appears slightly when only graphite is added; however, the plateaus are shown to extend across larger strain range for those composites loaded with binary nanofillers, regardless of clay to graphite ratio and total filler loading. The plateau is likely due to the close contact between clay and graphite, which leads to relative sliding between nanofillers under compressive forces (graphite is also a commonly known lubricant for mechanical systems). The van der Waals interaction between clay and graphite is weak, relative to the progressively increased compression loading. At a point where the binary nanofiller affinity cannot withstand the applied force, the interface would have to deform, leading to the observed slippage. Another possible explanation for this unique mechanical behavior could be that a small amount of water is trapped between the closely interacting filler layers, resulting in voids when freeze drying, which subsequently deform and yield a stress-strain behavior similar to Figure 6b. Similar explanations can be applied to the aerogels filled with only graphite when small plateau is noticed during compression.

**Figure 7:**
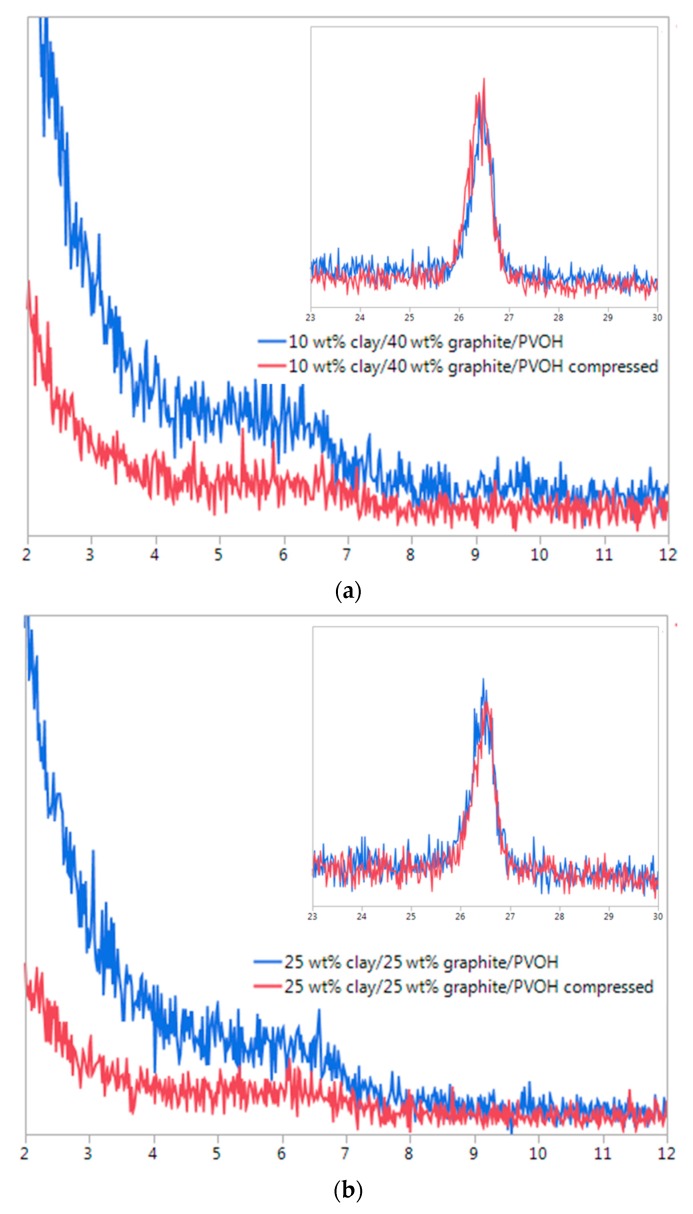
XRD spectra for 50 wt % clay/graphite/PVOH aerogels with 1:4 (**a**) and 1:1 (**b**) clay/graphite ratios before and after compression testing; intensity (au) vs. 2θ.

XRD was also used to monitor the structural changes of the composites before and after compression testing. Unlike some of the elastic aerogels where large amount of deformation could be recovered [24,25]. the rigid PVOH/clay/graphite composites exhibited good dimensional stability after mechanical compression, making it possible to study the compression-induced structural differences by XRD. As shown in Figure 7, no peak shift was noticed for graphite, relative to the pristine graphite powder, for the aerogels loaded with 50 wt % of nanofillers at either 1:1 or 1:4 clay/graphite ratios. The XRD patterns for graphite (002) plane were almost identical before and after mechanical compression for these aerogels. The composite containing 10 wt % clay and 40 wt % graphite showed a 2θ at about 6° before compression (Figure 7a), indicating a shift from the 2θ = 6.9° for the pristine clay and this value is also lower than the 2θ for clay-graphite aerogel without PVOH (Figure 3b). Although the XRD peak shift for characteristic clay (001) plane is not clearly identified comparing before and after compression, mechanical deformation led to a reduced magnitude for the clay peak in our opinion, which could likely result from sliding between clay and graphite layers. The impact of such mechanical deformation upon x-ray peaks of clay within composites has been previously reported [25]. Due to the affinity between clay and graphite, some graphite layers may be sandwiched (or partially sandwiched) between clay platelets, which were insufficient to exfoliate clay platelets. When the weak interface was destroyed by the compression stress and shear-induced deformation was introduced, more clay platelets would be forced to separate from large stacks. Similar clay exfoliation improvement was also observed for aerogel loaded with 1:1 clay/graphite ratio after compression (Figure 7b).

The influence on thermal conductivity of the aerogels filled with single or binary nanofillers were also tested. At 1:4 clay/graphite ratio, the composite containing 10 wt % clay and 40 wt % graphite showed a thermal conductivity of 0.182 ± 0.0049 W/mK, which is similar to the sample containing 2 wt % clay and 8 wt % graphite (0.183 ± 0.0042 W/mK), indicating that there is little change in thermal conductivity when the total filler loading was increased from 10 wt % to 50 wt %. The thermal conductivity results for 1:1 clay/graphite ratio samples are found to be similar to those with 1:4 clay/graphite ratio for total filler loading of 10 wt % or 50 wt %. The measured thermal conductivities are higher than typical PVOH aerogels, for example, thermal conductivity values as low as 0.025 W/mK have been previously reported for freeze dried PVOH/clay aerogels produced without graphite [25,26]. One possible reason for higher thermal conductivity is the high aerogel density, which was measured to be 0.12 g/cc for the sample containing 10 wt % clay and 40 wt % graphite. Besides porosity, other factors to take into consideration include nanofiller loading and aerogel morphology, such as layer thickness and orientation.

Electrical conductivities for the 50 wt % PVOH/clay/graphite aerogels with 1:1 and 1:4 clay/graphite ratios were also measured. Surprisingly, the electrical conductivity values for the composites with 1:1 and 1:4 ratios were 9.69 ± 0.34 × 10^−8^ S/m and 9.15 ± 0.42 × 10^−8^ S/m, respectively, which were significantly lower than other aerogels filled with conductive nanofillers such as carbon nanotubes [27]. At 1:1 clay/graphite ratio, the electrical conductivities were measured to be 11.14 ± 0.25 × 10^−8^ S/m and 9.50 ± 1.11 × 10^−8^ S/m for samples containing total filler loading of 20 wt % and 30 wt %, respectively. At such a low electrical conductivity range, the conductivity seemed not to be very sensitive to the clay to graphite ratio. The composites therefore exhibited very high percolation threshold, which reflected the onset of efficient network formation for conductive fillers (e.g., higher than 25 wt % and 40 wt % graphite for samples with 1:1 and 1:4 ratios, respectively). For the graphite-filled aerogel composites, the presence of large volume of air after ice sublimation would be beneficial for the electrical conductivity due to the excluded volume. However, longer conductive pathway may be needed due to parallel lamellar polymer framework, which potentially increases the contact resistance. Other factors likely to contribute to the low electrical conductivity of aerogels include polymer encapsulation after graphite dispersion, clay separation of graphite layers, graphite alignment and defects.

**Figure 8:**
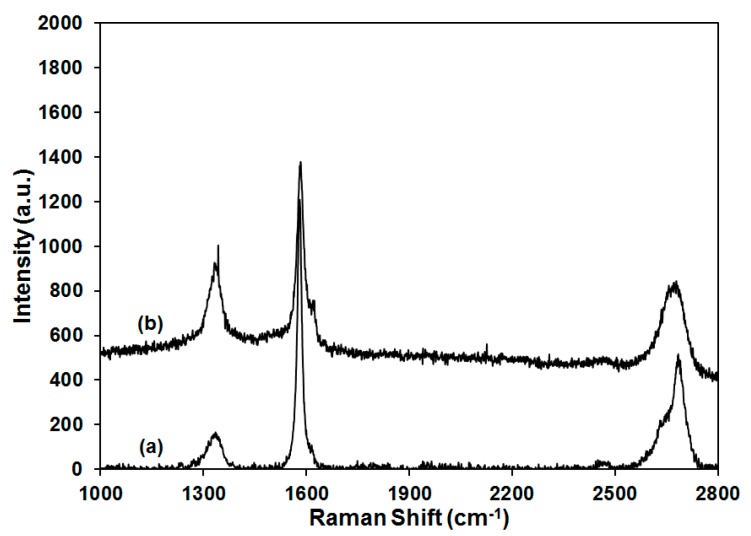
Raman spectra for pristine graphite (a) and PVOH aerogel containing 50 wt % graphite (b).

Raman as a useful tool to probe the functionalization and dispersion of graphite was also employed, as shown in Figure 8. The pristine graphite showed three characteristic peaks at ~1335 cm^−1^, ~1580 cm^−1^, and ~2680 cm^−1^, which represented the D band, G band and 2D band, respectively. The G band is related to E_2g_ vibrational mode of graphite layers, while the D band is responsible for the sp^3^ carbon content or structural defects on graphite. The ratio for the intensity of D and G bands (I_D_/I_G_) was normally used to determine the functionalization or defects of carbon nanotube or graphite, qualitatively [28]. The graphite used in this study was found to have I_D_/I_G_ value of 0.14, which is relatively high for as-received graphite, indicating the presence of some defect domains and in good agreement with the low electrical conductivity measured for the aerogels. When 50 wt % of graphite was introduced into PVOH aerogel, the I_D_/I_G_ value was increased to 0.48. Polymer inclusion and edge effects as a result of aerogel production are proposed to be responsible for the enhancement of I_D_/I_G_ value because no graphite functionalization was introduced during aerogel processing [29]. The 2D band is a second-order two-phonon mode, which is adopted together with G band for the study of graphite exfoliation. As shown in the insert of Figure 8, the 2D band for pristine graphite consists of two components (2D_1_ and 2D_2_), which are very close to the reported intensities (1/4 and 1/2 compared with the intensity of G band, respectively) [30]. For the PVOH aerogel containing 50 wt % of graphite, downshift of the 2D band was observed and compared with the Raman shift in the literature to estimate the number of layers for the graphite dispersion in PVOH [31]. According to the literature, graphite and graphene with 10, 5, 2, and 1 layers were labeled by the corresponding 2D band, showing a gradual transition of the Raman shift from ~2680 to ~2640 cm^−1^. Graphene with 5 layers exhibited significantly broadened 2D band relative to bulk material, but the peak at ~2680 cm^−1^ was clearly unchanged. When the number of layers was reduced to 2, the graphene showed a broad peak at ~2645 cm^−1^, which was close to the individual layer shift at ~2640 cm^−1^. In the current study, the pristine graphite showed a 2D peak at ~2680 cm^−1^ which shifted to ~2670 cm^−1^ for the 50 wt % graphite/PVOH aerogel, implying that the graphite was dispersed into graphene with less than 5 layers on average for the graphite/PVOH aerogel. Interference from clay prevented the recording of Raman spectra for aerogels containing both clay and graphite.

**Figure 9:**
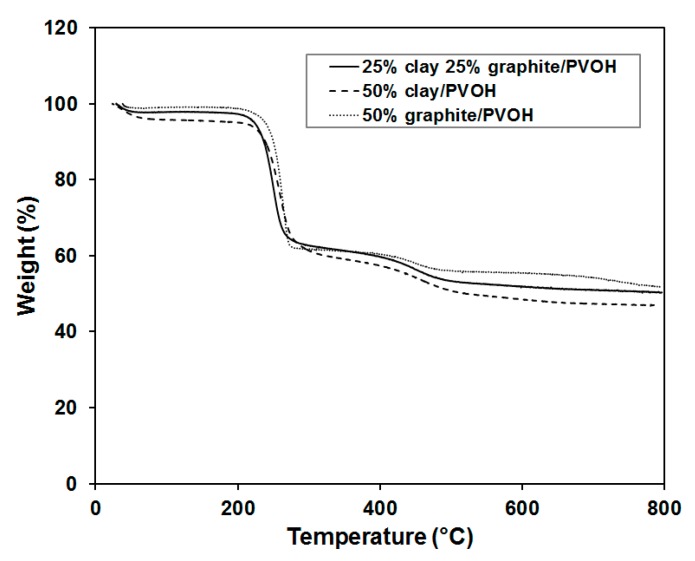
Thermogravimetric analysis (TGA) thermogram for PVOH aerogels loaded with 50 wt % of total nanofillers.

Thermal stabilities of the aerogels were characterized by thermogravimetric analysis (TGA), as shown in Figure 9. The transition temperatures and step pattern of the PVOH degradation closely match with those previously published for PVOH Aerogels [32]. The polymer aerogel containing 50 wt % of graphite showed the highest onset point for decomposition (*T*_0_), highest decomposition temperature (*T*_d_) and largest amount of residue at elevated temperature, which is consistent with strong interaction between graphite layers and PVOH. The sample loaded with 1:1 clay to graphite ratio exhibited the lowest *T*_0_ and *T*_d_, likely due to the affinity among clay and graphite. When PVOH was filled with just one nanofiller (clay or graphite), the nanofiller would be dispersed in the polymer to create significant amount of interfaces, which suppressed the mobility and entanglement of polymer chains. The introduction of second nanofiller, however, may not necessarily lead to stronger interactions with polymers for the reason that some nanoparticles tend to have good affinity with other nanoparticles (of the same kind or different kind), but not with polymers. The observation is also in good agreement with the results obtained by mechanical compression testing.

## 3. Conclusions

The use of clay as dispersing aid for the stabilization of graphite in water was demonstrated. A large quantity of graphite could be dispersed in water, presumably due to van der Waals interaction with clay. Aerogels produced from the suspensions showed lamellar structures composed of very thin layers of uniformly dispersed graphite and clay. When the binary nanofiller mixture (clay and graphite) was incorporated into PVOH aerogels, increased aerogel layer thickness and decreased interlayer spacing were observed, suggesting retardation of ice crystal formation on cold mold surfaces after nanofiller addition. A unique plateau region of the stress-strain curve was observed for aerogels filled with binary nanofillers, likely due to sliding between clay and graphite platelets under compressive load. Aerogels loaded with only graphite exhibited higher compressive modulus, yield stress and toughness, relative to the composites filled with binary nanofillers at the same total loading. Mechanical properties of the aerogels were insensitive to various clay/graphite ratios and synergistic effects were not observed for the filler loading range studied. XRD spectra of the aerogel showed slightly intercalated clay platelets when compared with the pristine clay and better clay exfoliation was also noticed after aerogel compression. Raman spectrum of the aerogel loaded with 50 wt % graphite strongly suggested that graphite was exfoliated down to graphene with less than 5 layers on average. This aerogel also exhibited higher decomposition temperature and char residue at elevated temperatures. Electrical and thermal conductivities remained low even after the addition of large amount of graphite, demonstrating the lack of an effective percolation pathway for phonons or electronics. Clay was found to have good affinity with graphite in water or PVOH aerogels, which may shed some light on formulation, processing and optimization of polymer nanocomposites.

## 4. Materials and Methods

### 4.1. Materials

Poly(vinyl alcohol) with a molecular weight of 31,000–50,000 g/mol (PVOH; Aldrich, St. Louis, MO, USA), synthetic graphite (batch #08017EH) (Aldrich) and sodium Montmorillonite (Na-MMT; PGW grade, cation exchange capacity (CEC) of 145 meq/100 g, Nanocor Inc. Arlington Heights, IL, USA) were used as received. Deionized (DI) water was obtained using a Barnstead RoPure reverse osmosis system.

### 4.2. Preparation of Clay and Graphite Suspensions and Aerogels

To produce clay/graphite suspensions, a total of 4 g clay and graphite was introduced into a glass vial, wherein 76 g of water was subsequently added. Different ratios of clay to graphite were used but the total solid concentration was kept at 5 wt % for all the suspensions (for example, a ratio of 1:1 means the addition of 2 g of clay and 2 g of graphite). The liquid mixture was sonicated for three hours in a bath sonicator (BRANSON 2510, Branson Ultrasonic Corp., Danbury, CT, USA) and stirred overnight to produce clay/graphite suspensions. To demonstrate the dispersion of graphite, a liquid drop was taken right after sonication for each of the suspensions and put on a glass slide to be imaged using a digital camera (Canon PowerShot SD850 IS, Canon Inc., Tokyo, Japan). The clay/graphite suspensions were then frozen by immersing the vials into the ethanol/solid CO_2_ (s) cooling baths. The samples were freeze dried using a Virtis Advantage EL-85 lyophilizer with shelf temperature of 25 °C, condenser temperature of −80 °C and an ultimate vacuum of ~5 µbar.

### 4.3. Preparation of Poly(vinyl alcohol) (PVOH) Suspensions and Composite Aerogels Containing Clay and Graphite 

The PVOH was first dissolved in DI water at 90 °C to produce a 5 wt % solution. Then appropriate amount of clay and graphite was introduced into the PVOH solution so that the total solid concentration of the two fillers in dried polymer composites varied from 10–50 wt %. Three clay to graphite ratios were studied (1:1, 4:1, and 1:4) to differentiate the impact from the two fillers. For example, a 10 wt % suspension with clay to graphite ratio of 1:4 would contain 9 g PVOH (in 180 g of solution), 0.2 g clay and 0.8 g graphite. The liquid was mechanically stirred in a Waring laboratory blender at high speed (~22,000 rpm) for 2 min and low speed for 4 min. One hour of water bath sonication was applied subsequently and the suspension was vigorously stirred at 60 °C for three hours. The PVOH/clay/graphite aerogels were fabricated using the same process as described above for the clay/graphite aerogels.

### 4.4. Characterization

SEM imaging and spectral mapping analysis were carried out using FEI Quanta 3D 200i FE-SEM for the samples sputter coated with a thin layer (~5 nm) of palladium. Mechanical compression tests were conducted by an Instron model 5565 universal testing machine fitted with a 1 kN load cell operating at a crosshead speed of 1 mm/min. At least four cylindrical specimens from each composition with ~20 mm diameter and height were tested. The compressive modulus and toughness values were calculated based on the slope of the elastic region and the integrated area under stress-strain curve at given strain, respectively. Thermal stability of the aerogels was examined using a TGA Q500 (TA Instruments) from 25 to 800 °C at 10 °C/min. XRD spectra were recorded by Rigaku diffractometer (RINT 2000 series) with CuKα source (1.5418 Ǻ). Raman spectra were collected using Horiba LabRAM with 633 nm excitation and 100× objective. The aerogel samples were cut into a slender cross-section (typically ~30 mm × 8 mm × 1 mm) for electrical conductivity measurements, and into a coin shape (12.7 mm in diameter and 3 mm in thickness) for thermal conductivity measurements. The pieces with coin shape were cut from one aerogel sample to minimize errors due to the variation between samples. Electrical resistance was obtained from linear slopes of current-voltage (I-V) sweeping measurement result between −10^−4^~10^−4^ A along the long edge for samples painted with four electrode lines. The electrical resistivity was calculated by multiplying geometrical factors to the electrical resistance. The thermal conductivity of the sample was measured by a homemade ASTM D5470 apparatus wherein the aerogel samples were mounted between two rods with a silicone heat sink compound [33]. Temperature gradients were made along the rods and temperature drops across the out-of-plane direction of the coin shape sample were measured to obtain thermal conductance. The heat fluxes through the two rods were measured to be within 10%. The thermal conductivity of the aerogel samples was calculated by considering the apparent volume (i.e., including the air as a part of the sample).
